# Patient survey of value in relation to radiology: results from a survey of the European Society of Radiology (ESR) value-based radiology subcommittee

**DOI:** 10.1186/s13244-020-00943-x

**Published:** 2021-01-07

**Authors:** European Society of Radiology (ESR), Michael Fuchsjäger, Lorenzo Derchi, Bernd Hamm, Adrian P. Brady, Carlo Catalano, Andrea Rockall, Peter Mildenberger, Guy Frija, Marc Dewey, Thomas Kröncke, Judy Birch

**Affiliations:** Am Gestade 1, 1010 Vienna, Austria

**Keywords:** Value-based healthcare, Radiology, Patient empowerment

## Abstract

**Rationale and methodology:**

A survey of patients was carried out between January and June 2019, to better understand how patients interpret value in relation to radiology as a means to refining the concept of Value-Based Radiology (VBR) in Europe, ensure radiology’s value is properly weighted in Value-Based Health Care (VBH) metrics, and maximise the value of radiological services to patients. The survey was disseminated via various heads of radiology departments, ESR officers, patient organisations, and ESR website and social media channels.

**Results:**

Responses were received from 400 patients from 22 countries. Whilst most expressed general satisfaction with the radiological services they received, certain aspects of the radiological services they received left room for improvement. Thirty-six percent of respondents reported that they were not satisfied with the information provided about the risks and benefits of procedures, and thirty-three percent reported not being satisfied with the availability of radiologists for consultation, potentially suggesting that some patients lack sufficient information to participate fully in treatment decisions. Patients were often unaware of what information they were entitled to receive. Over eighty percent of respondents were unfamiliar with the concepts of Value-Based Radiology and/or Value-Based Health Care.

**Conclusion:**

In addition to procedural correctness (correct diagnosis, appropriate procedures performed), patients highly value information and communication with their radiologist (information provided about procedures, explanation of results, personal consultation). Lack of communication was found to be a cause of dissatisfaction in many cases. This could suggest a means of improving patient outcomes as measured by Value-Based Health Care metrics.

## Key points

- Patients are currently unfamiliar with VBHC and VBR concepts.
- There is general satisfaction with radiology services.
- Appropriately performed examinations and correct diagnoses are key to patients’ conception of value.
- Insufficient communication is a significant cause for patients’ dissatisfaction.
- Simple measures could have a significant impact in improving communication and patient satisfaction.

## Patient summary

An ESR survey was carried out among patients in 2019, to better understand how they perceive the value of radiology to improve the concept of Value-Based Radiology (VBR) in Europe, ensure radiology’s value is properly weighted in Value-Based Health Care metrics, and maximise the value of radiological services to patients.

Whilst most expressed general satisfaction with the radiological services they received, certain aspects left room for improvement. The responses from 400 patients based in 22 countries highlight that 80% are unfamiliar with the concepts of VBR and Value-Based healthcare, which seeks to place quality at the centre of the healthcare decision-making to reduce costs and improve health outcomes by providing patient-centred services.

However, radiology is often overlooked and included only as a cost in the healthcare value chain although the ESR strives to show the crucial role of radiology in contributing value to many patients.

The results of the survey showed that the lack of communication was the main cause of dissatisfaction. Indeed, the satisfaction with the information provided pre-procedure and the availability of the radiologist had the lowest average ratings, and half of respondents were unaware of their right to receive information on radiation dose exposure. Also, it underlined that patients considered that the three most important aspects when receiving radiology services were the absence of errors in the diagnosis, the performance of the appropriate procedure and a swift diagnosis.

The outcomes of the survey highlighted that improved communication and changes to direct radiologist-patient communication could boost the perceived value of radiological services, but would require much greater investment in resources.

## Introduction

Value-based health Care (VBHC) is a concept initiated by the pioneering work of Harvard economist Michael Porter [1], which sought to place quality, rather than quantity, at the centre of healthcare decision-making in an attempt to simultaneously reduce costs and improve health outcomes. This was a response to the increasing costs of healthcare provision, especially in developed countries, today´s healthcare focus on the treatment of acute and emergency episodes, values fee for service models and provides little incentives for investment in “prevention, longitudinal chronic disease management, [or] population health” [2]. VBHC is an attempt to provide a different perspective and re-locate patients at the centre of healthcare. Porter’s conception of value (patient health outcome divided by money spent) would suggest two ways in which value to patients may be increased: either reducing costs for the same outcome, or increasing outcomes relative to costs. However, the metric used by Porter for assessing value, specifically, health outcome over money spent, has been criticised as lacking nuance. The well-known Utah Value in Health Care Survey defined value as the “product of the quality of care *plus the patient experience* at a given cost” (emphasis added) [3]. Thus, a subjective element was incorporated within the value equation by recognising the importance of the patient’s assessment of value.

The notion of including patients’ perspectives has long been integral to the ESR’s Value-Based Radiology Subcommittee: the ESR was a pioneer amongst medical scientific societies in creating a Patient Advisory Group (ESR-PAG) within the society structure, with the explicit aim of drawing together “patients, the public and imaging professionals in order to positively influence advances in the field of medical imaging to the benefit of patients in Europe” [4], and an ESR-PAG representative is purposefully included within the Value-Based Radiology subcommittee.

The European Commission established a ‘multisectoral and independent expert panel to provide advice on effective ways of investing in health’ in 2012. This expert panel produced a draft opinion on VBHC in May 2019, whilst this survey was in progress. The expert panel concluded that “available resources—not only financial but also in terms of time—are finite so it is essential that patients and clinicians get the greatest value from what is available.” [5]. The expert panel proposed four metrics for the measurement of value: personal value (“appropriate care to achieve patients’ personal goals”), technical value (“achievement of best possible outcomes with available resources”), allocative value (“equitable resource distribution across all patient groups”), and societal value (“contribution of healthcare to social participation and connectedness”) [5].

To date, radiology’s place in the value chain has remained, to a large extent, overlooked: radiology is either omitted from the value chain, or included only as a cost: “radiology is widely viewed as a contributor to health care costs without an adequate understanding of its contribution to downstream cost savings or improvement in patient outcomes.” [6]. As the Utah Survey makes clear, the improved patient outcomes carry a subjective element which must also be understood in order for radiology to be properly valued within the whole healthcare value chain. Swift and accurate diagnosis is crucial in determining the ability to meet patients’ needs successfully. This was also recognised in Porter’s initial conception: “Delays in diagnosis or formulation of treatment plans can cause unnecessary anxiety” [1]. Anxiety would, without doubt, negatively affect value of the service received under the subjective element of the University of Utah Health’s framework. In addition, the fact that erroneous diagnosis can lead to reduced health outcomes, through both failure to treat disease optimally and the unnecessary performance of inappropriate procedures, hardly needs explanation. In 2017, the ESR published a position paper on Value-Based Radiology, exploring the crucial role of radiology in contributing value to many patients [7]. More recently, the ESR and other societies have published a ‘call-to-arms’ on Value Based Radiology in the Journal of the American Medical Association, seeking to engage other specialties in cooperative measures to enhance value creation by radiology for the benefit of patients [8].

As, in the future, planning and resource allocation will almost inevitably depend on value-based metrics, it is of the upmost importance to make certain that radiology’s contribution to achieving optimum value is recognised. For this reason also, it is vital to understand how patients perceive the value of radiology (in line with the first pillar of the European commission’s expert panel’s draft report) and how that value to patients may be maximised. On this basis, the ESR Value-Based Radiology Subcommittee implemented a survey in January 2019 with the goal of questioning patients about what aspects of the radiological services they received they valued highly and, potentially, where value could be increased. The results of this survey may contribute to assessing the ways in which patients perceive value in relation to the provision of radiological services.

## Materials and methods

### Survey design

As the survey was designed to assess subjective values, in order to standardise responses, a rating scale of 1–5 was employed, where 1 was ‘very unsatisfied’ and 5 was ‘very satisfied’. This survey was separated into sections that covered: (1) basic demographics such as geographical location (country), age, and gender; (2) basics of the patient’s medical history (e.g. number of times they had undergone radiological scans in the preceding 2 years) and their satisfaction in relation to care received (e.g. rating different aspects of the radiology service provided, such as courtesy of staff, information provided about benefits and risks of the procedure, waiting times etc.); (3) questions related to the patient’s familiarity with the concept of value-based radiology and their general attitudes towards value (e.g. what they consider the most important aspect of value: cost, efficiency/safety, or service); and, (4) questions related to how patients assess specific aspects of value in relation to radiological services (e.g. what factors they value most highly when receiving radiological services).

The survey was conducted between 29th January and 28th June 2019. The survey was developed with the assistance of the ESR’s Value-Based Radiology Subcommittee. The full printable version of the survey can be found on the ESR website (https://www.myesr.org/esr-patient-survey-value-based-radiology). Furthermore, the English version is included as an annex to this article (Additional file 1).

### Survey administration

The ESR Board of Directors, members of the ESR Value-Based Radiology Subcommittee, members of the EuroSafe Imaging Stars Network, and representatives of the ESR-PAG were sent the survey invitation on 29th January 2019, with a request that this be distributed to patients who had undergone radiological examinations and as many surveys as possible be returned by 15th February in order to allow preliminary results to be presented at the European Congress of Radiology (ECR) 2019. The survey was further disseminated via social media channels, both by the ESR and the ESR-PAG and its contacts. In order to capture as broad a range of patient opinions as possible, Bulgarian, Dutch, English, French, German, Italian, Russian, and Spanish versions of the survey were produced. The survey was also made available as a printable document which could be filled in by hand and returned to the ESR office. Preliminary results were presented during the Value-Based Radiology Coffee & Talk Session at the ECR 2019; however, the survey remained open for a total of five months, closing on 28th June 2019.

The survey was conducted using both an online tool (Survey Monkey™) and a printable version of the survey which could be filled out by hand and returned to the ESR office. Links to the online version of the survey were provided on the ESR website.

### Analysis

The online results in each language were downloaded individually and combined with the results of paper surveys to produce a final set of data for analysis (Additional file 1). Summary statistics were generated using Microsoft Excel. Average scores were calculated (e.g. where respondents rated their satisfaction on the scale of 1–5), which could then be interpreted. Percentages were also calculated to allow for standardisation as, for some questions, the number of responses varied. This variation in response number also provided useful information for interpretation.

## Results

### Basic demographics

**Fig. 1:**
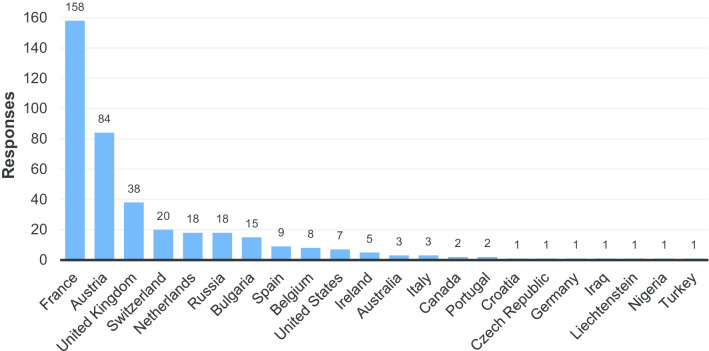
Responses by country

Following the closure of the survey, all responses were collated. The final number of responses was precisely 400, representing 22 different countries (Fig. 1). Partial responses to the online survey were received from respondents from other countries but, as they were substantially incomplete, these were discarded for the final analysis.

**Fig. 2:**
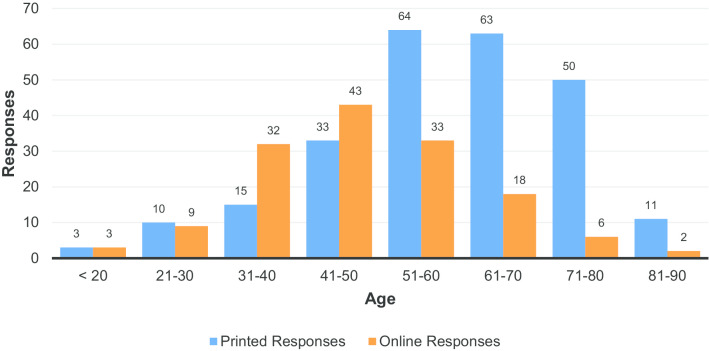
Responses by type of response, stratified by age

**Fig. 3:**
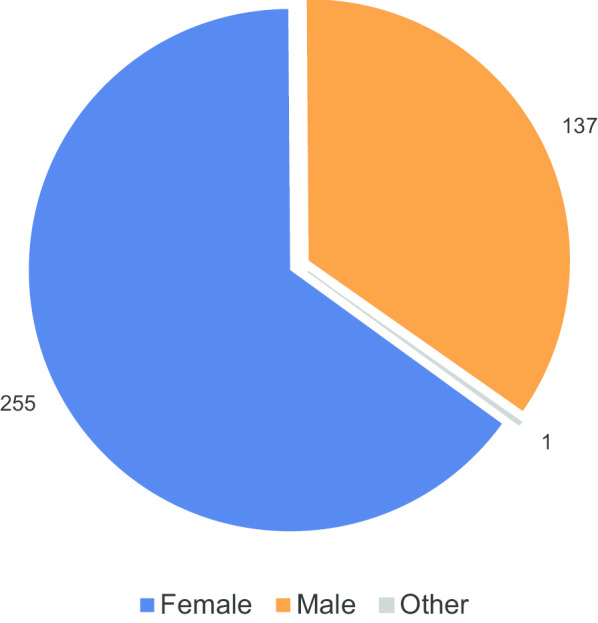
Gender of respondents

Figure 2 shows the breakdown of respondents according to age. These figures show that online respondents were, on average, younger than those that responded to printed surveys provided to them in their hospitals: over 50% of online respondents were under 50, whereas less than 25% of respondents to the written survey were under 50. Approximately two-thirds of respondents were female (Fig. 3).

### Basics of the patient’s medical history and their satisfaction in relation to care received

**Fig. 4:**
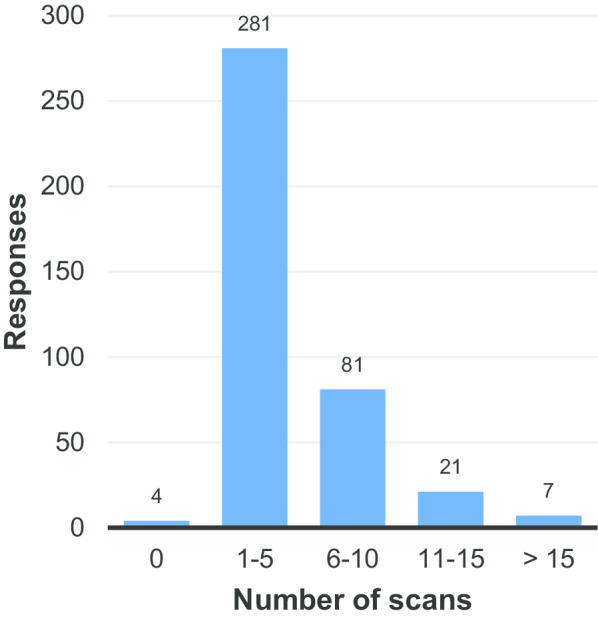
How many times have respondents undergone radiological scans in the last 2 years?

Figure 4 reveals that most respondents had undergone between 1 and 5 radiological procedures in the preceding 2 years. The fact that four respondents reported not having undergone any radiological procedures in the last 2 years (Fig. 4) is curious, but potentially explicable by the fact that they had undergone such procedures at an earlier date.

**Fig. 5:**
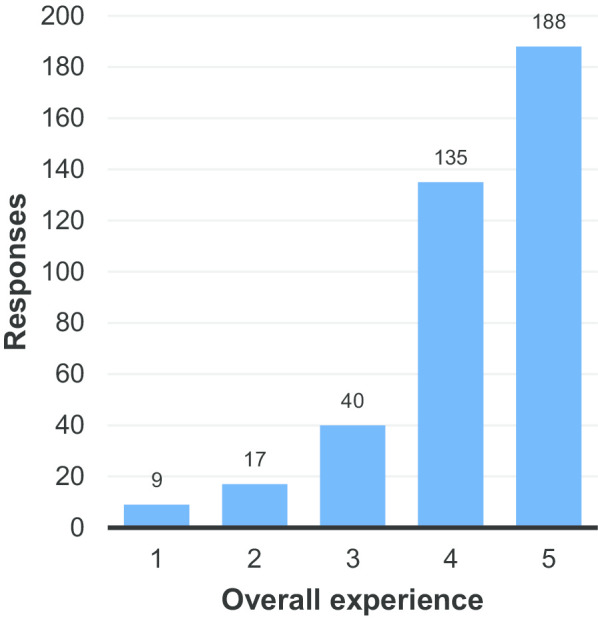
Satisfaction of respondents with the overall experience with radiological services in the last 2 years (1 = very unsatisfied, 5 = very satisfied)

Figure 5 shows the overall satisfaction of respondents with the quality of the radiological services which they received. The average rating of 4.22 (Additional file 1: Table S5) is slightly higher than in the preliminary results (4.17), but can be considered broadly consistent with expectations based on the preliminary results. Whilst there remains room for improvement, the vast majority of respondents reported being satisfied or very satisfied with the service received.

**Fig. 6:**
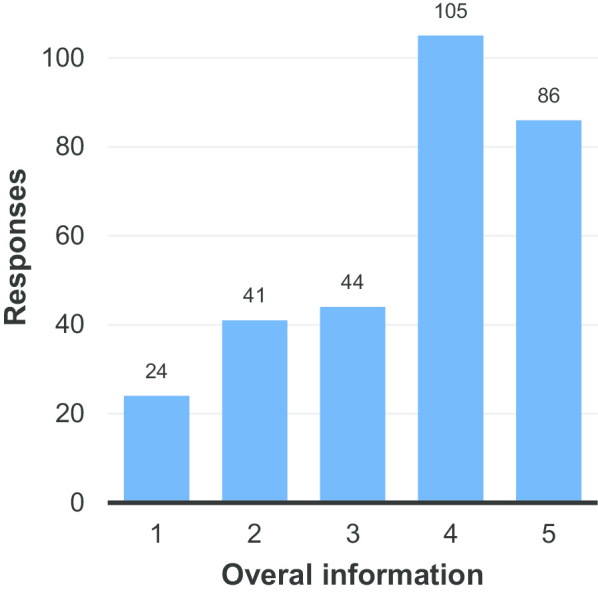
Satisfaction of respondents with the information provided about the procedure, e.g.risks, benefits, description of how it is performed (1 = very unsatisfied, 5 = very satisfied)

Figure 6 again shows an increase in average patient satisfaction with the information provided prior to procedures compared to the preliminary results (3.63 compared to 3.47—Additional file 1: Table S6). Perhaps more noticeable however was the large number of respondents who skipped this question (25%—see Additional file 1: Table S6).

**Fig. 7:**
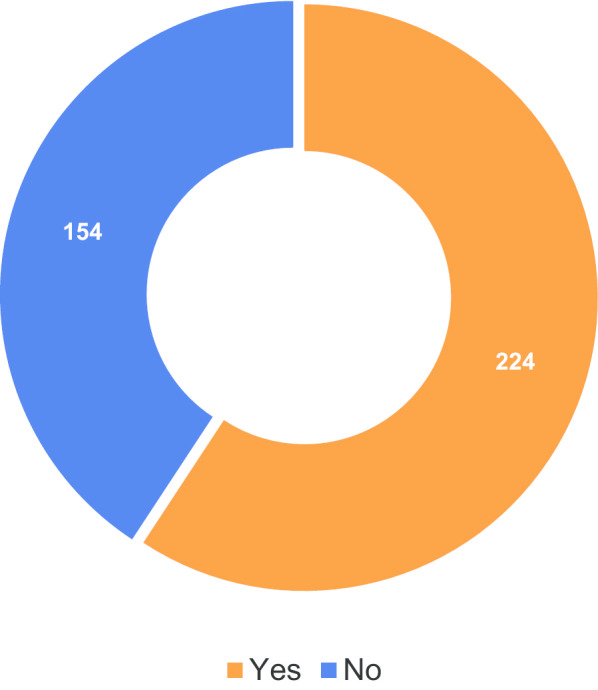
Respondents who were supplied a copy of the radiology report from their examination

**Fig. 8:**
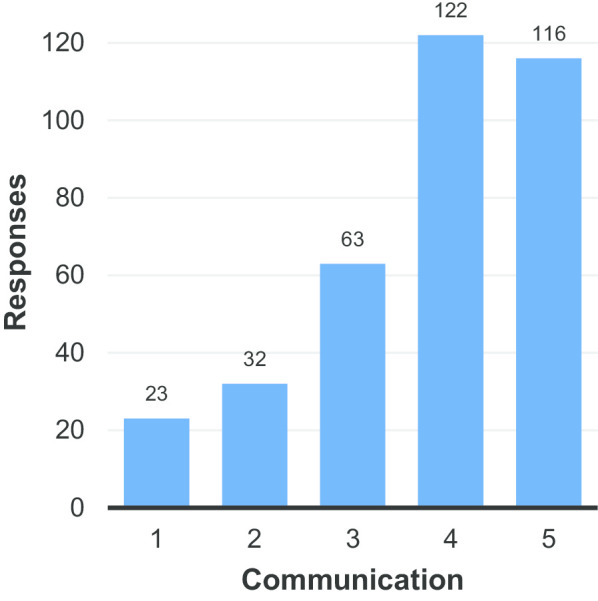
Satisfaction of respondents with the communication of results (1 = very unsatisfied, 5 = very satisfied)

Figure 7 shows the number of patients who reported being supplied with a copy of their radiology report. Figure 8 shows the level of satisfaction in the way in which results were communicated; but, again, a significant number of respondents skipped this question (Additional file 1: Table S8).

**Fig. 9:**
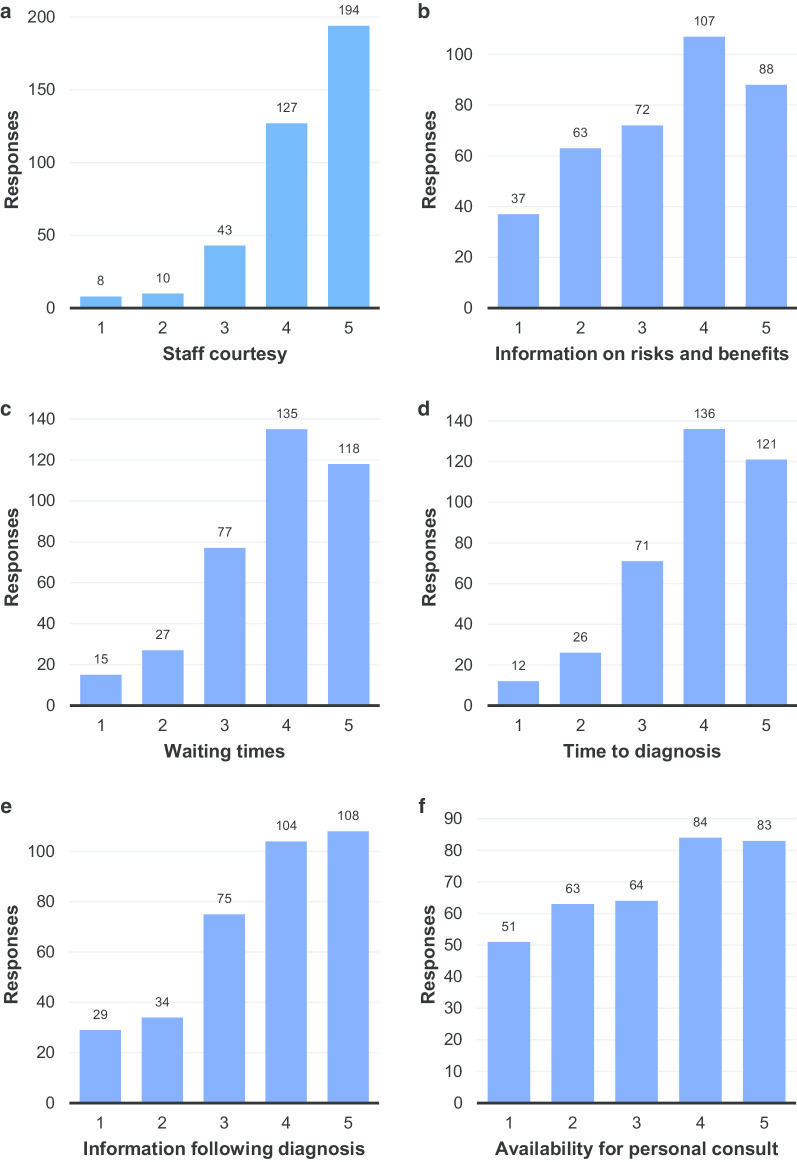
Rating of the following aspects of the radiology service: **a** Courtesy of the staff. **b** Information provided about benefits & risks of the procedure. **c** Waiting times (i.e. from referral to appointment). **d** Time-to-diagnosis (from initial referral). **e** Information provided by radiology staff following diagnosis. **f** Availability of the radiologist for personal consultation. (1 = very unsatisfied, 5 = very satisfied)

Figure 9a–f shows satisfaction with various aspects of the radiology service. The only aspect with which substantial satisfaction was expressed was the courtesy of the staff (Fig. 9a). Satisfaction with the information provided pre-procedure and the availability of the radiologist for personal consultation had the lowest average ratings with 3.40 and 3.25 respectively (Additional file 1: Tables S9B and S9F), though the latter was still substantially higher than in the preliminary results (2.02). These two aspects were also the only two in this section to have fewer than 200 respondents (i.e. 50%) rate their level of satisfaction as four or five. The next lowest average rating was for the information provided post-diagnosis (Additional file 1: Table S9E). Figure 9b, e, f were the only ones in which more than five percent of respondents answered that they were ‘very unsatisfied’ with the service provided (Additional file 1: Tables S9B, S9E and S9F). The latter was the only aspect of the radiology service for which more than 10% of respondents answered that they were ‘very unsatisfied’.

These results suggest that, whilst all aspects of the radiology service could be improved, communication with patients is an area that is in particular need of attention.

### Questions related to the patient’s familiarity with the concept of value-based radiology and their general attitudes towards value

**Fig. 10:**
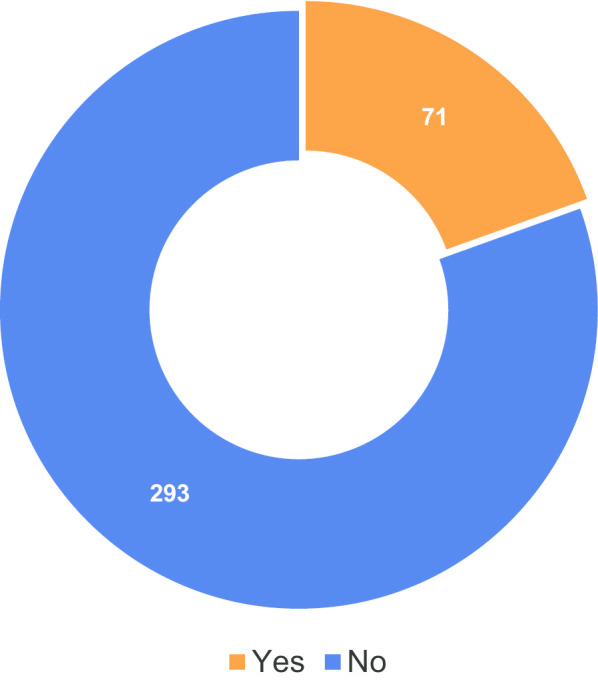
Respondents previously familiar with the concept of value-based healthcare (VBH)/value-based radiology (VBR)

The next question on the survey (question 12) informed patients that “Value-based care is a philosophy of healthcare achieved when professionals intentionally consider the quality of care provided, and the overall outcomes of that care, in relation to cost-efficiency” and asked them if they were previously familiar with either the concept of Value-Based Healthcare or Value-Based Radiology. Figure 10 illustrates that most respondents were not familiar with either concept.

**Fig. 11:**
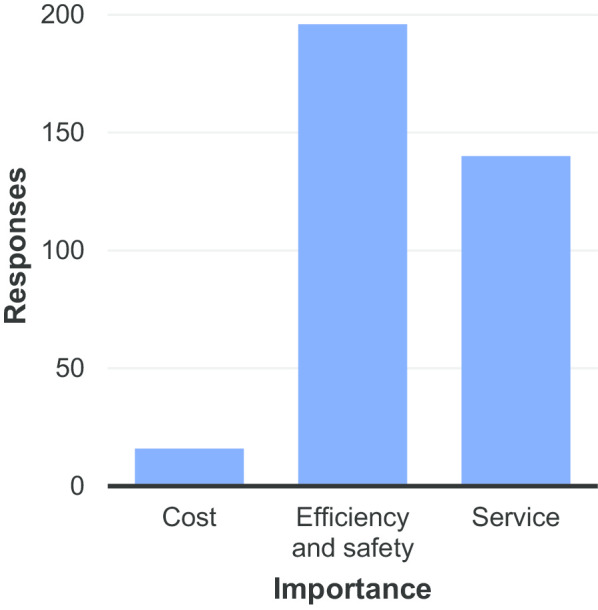
Perceived most important aspect of value in radiology

The subsequent question, question 13, asked what aspect of value respondents consider most important in radiology. The results are displayed in Fig. 11.

### Questions related to how patients assess specific aspects of value in relation to radiological services

**Fig. 12:**
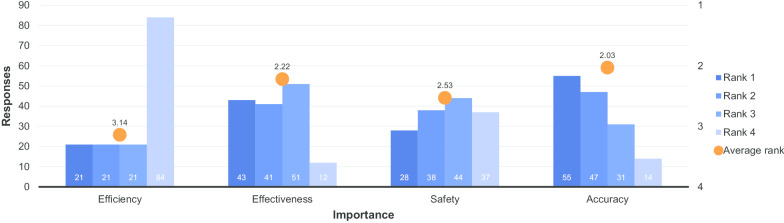
Quality aspects considered most important in radiology (1 = most important, 4 = least important)

**Fig. 13:**
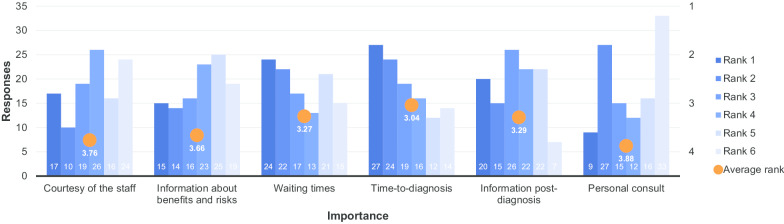
Service aspect considered most important in radiology (1 = most important, 6 = least important)

Those who selected ‘Efficiency/Safety’ as their most important aspect of value were asked to refine this by ranking different aspects of efficiency/safety according to their importance (1 = most important, 4 = least important, a ranking scale, as opposed to the numerical/rating scale used in earlier questions.). Figure 12 shows the outcome of this. Figure 13 shows the outcome of question 16, which asked those that responded ‘Service’ in question 13 to rank various aspects of service according to their importance (1 = most important, 6 = least important).

**Fig. 14:**
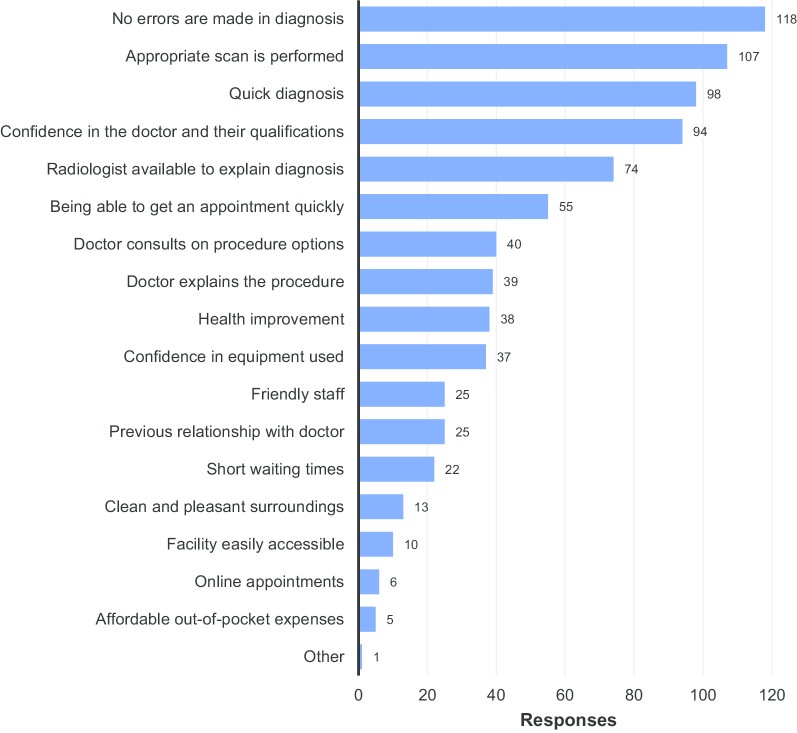
Factors considered most important in receiving radiology services

Figure 14 displays the factors respondents considered most important when receiving radiology services. The absence of errors in the diagnosis, the performance of the appropriate procedure, and a swift diagnosis were the most frequently chosen in respondents’ top three. The respondent that answered ‘other’ wrote in “accuracy of the report” and cited many errors in their report, suggesting, in fact, that this also should have been counted as ‘No errors are made in diagnosis’.

## Discussion

The online tool allowed logic to be applied to the survey, i.e. depending on the answer given to a certain question, different sub-questions would be revealed. Consequently, not all questions in the survey were presented to all online respondents. Conversely, in the printable version of the survey, all questions/sub-questions were displayed along with instructions to go to different sections depending on the answer given to particular questions. However, during analysis it became clear that not all respondents followed these instructions. As a result, the printable surveys sometimes contained answers that would have been invalid or impossible in the online version. In addition, not all questions on the online survey were mandatory; thus, some respondents did not answer all the questions with which they were presented. This was also true of the printable survey: some respondents returned incomplete surveys. This was an inevitable result of the attempt to capture data from the broadest possible range of respondents by providing both online and manually-completable versions of the survey and does not necessarily indicate an intrinsic flaw in the study design. For these reasons, there is a degree of variance in the number of responses received to different questions. Nevertheless, useful data was obtained.

Further difficulty was evident in one written survey in which the respondent answered ‘1’ (very unsatisfied) for all the questions in Fig. 9a–f, then added in comments ‘ich bin zufrieden’ (I am satisfied), indicating they had mixed the scale up. This probably was not an isolated incident and may be attributed to cultural differences (in the German-speaking education system a grade of ‘1’ would indicate high achievement, whereas ‘5’ would indicate unsatisfactory work—the inverse of the grading system in the Anglophone world). In any follow up survey, this issue might be addressed by using a pictorial scale (e.g. happy/unhappy faces) rather than a numerical scale.

As can be seen in Fig. 1, over 50% of responses came from two countries (France and Austria). The vast majority of these were paper surveys that were returned by two institutions: the Radiology Department of the Hôpital Beaujon in Clichy, France, and the University Clinic for Radiology at the Medical University of Graz, Austria (the institution of one of the authors). The lack of diversity is at least partly attributable to the fact that in some countries (e.g. Germany) it was reported that difficulties were encountered in gaining approval from hospital boards to distribute surveys to patients. Therefore, the results may not be fully representative given the low response rate from many of the countries involved. The total number of responses was, of course, small relative to the number of radiological procedures carried out across Europe. However, even small datasets may provide useful information, such as demonstrating that patients continue to have a poor understanding of the concept of VBR (Fig. 10). Additionally, the data that was collected confirms that the survey was worthwhile as it does reveal areas in which further investigation might prove beneficial. Should the study be repeated, agreements could be made with targeted hospitals or healthcare authorities for circulation in different institutions/countries/regions before the survey is released in order to ensure greater response rates and an even broader distribution of responses.

A major limitation is that it was not possible to see which respondents come from countries which have public health systems and which respondents were from countries with private systems as this could have generated differences in the answers.

The numbers in Fig. 2 reveal a clear difference between the demographics responding to the online and printable surveys. This suggests that the methodological decision to use both online and printed versions of the survey was justified as it enabled a broader picture to be captured.

It is speculated that the number of respondents who failed to provide an answer to question 5 (Fig. 6) could reflect their not being provided with any information about their procedure. Figure 7 certainly confirms that not all patients appear to have been given the appropriate information about their procedure: almost 40% denied receiving a copy of the radiology report from their examination. It should be noted that on the printed surveys, some respondents wrote in the answer ‘not always’ for this question. For the purposes of analysis, these were counted as ‘no’. The number of respondents not receiving reports for all their examinations could also explain the relatively high number of respondents that did not answer this question. Certainly, it appears that, in terms of ensuring patients are properly informed about procedures they have undergone, there is a degree of inconsistency in practice that could be improved upon.

It is also speculated that the high number of respondents skipping question 7 (Fig. 8) and the ambivalent responses (average rating: 3.78) could be linked to the number of respondents reporting not receiving radiology reports for their examinations.

With regards to Fig. 9b, it should be noted that reducing anxiety is a way in which radiologists can easily provide value to patients at little cost (e.g. explanatory posters in patient waiting areas, leaflets explaining procedures, information on departmental websites). In an era in which (mis)information is easily available to patients online, it is very important that radiologists provide clear and accurate information to their patients. Indeed, it is suggested that the responses in Fig. 9b, e, f (satisfaction with: information provided on risks and benefits of the procedure; information provided by radiology staff following diagnosis; and, the availability of the radiologist for personal consultation) could all be improved with changes in communication practices, which could potentially have a substantial impact on patient satisfaction and, therefore, their perception of the value of radiological services.

9B and 9E (satisfaction with: information provided on risks and benefits of the procedure and information provided by radiology staff following diagnosis) could potentially be improved with relatively simple and inexpensive changes, however, for 9F (the availability of the radiologist for personal consultation), the implementation of changes would require substantial changes in the organisation of resources in radiology departments to make time available for consultation with patients. Whilst organisational efforts are directed towards diagnostic quality (correct diagnoses; good-quality studies) and efficiency (the highest number of examinations possible per unit of time), such changes cannot be considered trivial. One potential means to achieving such changes would be an alliance between radiologists and patients both asking (together) to have a third departmental goal: communication availability. However, even a limited increase in communication could prove to be useful in improving patient satisfaction in this area. The impact of increased numbers of teleconsultations in the COVID-era is a potential area for further study.

For question 13 (Fig. 11), the online version of the survey only allowed respondents to select one option. However, on the printed version, despite instructions to select only one aspect, a number of patients selected multiple options. The difficulty in choosing (and perhaps the unfamiliarity with the concept revealed in Fig. 10) is perhaps the reason why so many respondents skipped this question. As we would expect in Europe (where countries are committed to achieving universal health coverage (UHC), “reinforced through the adoption of the United Nations (UN) Sustainable Development Goals (SDG), Goal 3 on health and the UHC target therein” [5]), costs were not considered an important aspect of value in radiology. ‘Efficiency/safety’ was considered the most important aspect of value in radiology by the greatest number of respondents. Indeed, so few respondents reported cost as the most important factor to question 13, that the results to the following question (question 14) did not provide useful data for analysis.

As with earlier questions, the online survey was able to apply logic to the responses and to direct respondents to different questions depending on their answers, which was not possible in the printed survey. Although instructions were provided alongside each option in the printable version of the survey (e.g. ‘Efficiency/Safety (go to part 5)’), these instructions were not always followed correctly (perhaps also due to respondents, in some cases, selecting multiple answers). This had an impact on the number of responses to question 15 (for those who responded ‘Efficiency/Safety’ in question 13) and question 16 (for those who responded ‘Service’ in question 13).

In total 269 valid responses were received to question 21. Significant numbers of invalid responses were received for this question in the printed surveys (131 respondents either skipped the question entirely or gave an invalid answer, e.g. by selecting more than three options). Again, the ability to programme the online survey prevented this occurring in the online responses. However, the responses that were collected broadly provided support for the conclusions reached above.

## Conclusions

This survey was conducted with the aim of gathering information on how the value patients ascribe to the radiology services they receive might be maximised, with a view to ensuring radiology is properly recognised as a contributor to value within the VBHC value chain.

Whilst this study has limitations (as further laid out in the discussion), useful data was collected. The responses to the survey, although relatively limited in number, revealed that improved communication with patients could have a significant impact on patients’ outcomes, for example by reducing their anxiety and allowing them to participate more fully in treatment decisions. Patients were clearly not familiar with the concept of VBHC, something that obviously needs to be addressed as healthcare moves towards this metric in order to ensure patients are not excluded and that all four pillars identified by the European Commission’s expert panel are fully realised.

It is believed that this survey has actionable findings for uptake by the radiology profession: with relatively minor adjustments (see suggestions for explanatory posters, leaflets etc. below), it would appear that patient satisfaction could be increased. Improved communication appears to be a high-impact way to boost the perceived value of radiological services. Wider-ranging changes in direct radiologist-patient communication would also improve patient satisfaction, but would require much greater investment of resources.

The survey also revealed a degree of ignorance on the part of patients as to exactly what information they are entitled to receive: 194 respondents reported that they were not aware that, after undergoing an X-ray or CT procedure. information regarding their radiation exposure should form part of the report which they are entitled to receive under article 58.b of the Basic Safety Standards Directive [9] (question 8). A further 37 respondents skipped question 8. Although the skipped questions could also have been due to not all respondents having undergone X-ray or CT procedures (the questions related to the entitlement to receive information about radiation dose, which only applies to modalities employing ionising radiation), the results suggest that over half of the respondents were unaware that they were entitled to receive information about their radiation dose exposure.

Based on the responses that were received to questions 15 and 16 (displayed in Figs. 12, 13, respectively), for those who chose ‘efficiency/safety’ as the most important aspect of value, the accuracy/correctness of the diagnosis clearly played the greatest role in their perception of the value of the service they received. The number of procedures they had to undergo to get the right results was of least concern to them. For respondents that chose the quality of the service as the determining factor in value, the time until they received the diagnosis was ranked as the most significant factor in determining value. The availability of the radiologist for consultation was ranked as the least important feature of good service, which, interestingly, contrasts somewhat with the dissatisfaction displayed in Fig. 9f and the importance given to it in Fig. 14 (where it gained the fifth most responses).

Question 21 on the survey (Fig. 14) revealed that procedural correctness (in terms of correct diagnosis and appropriate procedure being performed), swift results, and confidence in the doctor and their qualifications were the most highly valued factors in receiving radiological services. However, consultation on procedure options, explanations of procedures to be undergone, and the availability of the radiologist to explain the diagnosis all received more votes than ‘my health improves’. This again underscores the importance and value of communicating clearly with patients.

In conclusion, it is suggested that the data collected in this survey reveals both the need for improved communication with patients and the potential utility of a broader and more comprehensive investigation of patient experience of VBR and how value could be maximised. Certainly, the data collected in this survey can serve as a useful starting point for the refinement of more targeted questions. It is further suggested that, in any follow-up survey, the methodology should be adjusted to address the issues revealed in the discussion below.

## Supplementary information

**Additional file 1.** English version of the survey and final set of data for analysis.

## Data Availability

Not applicable.

## Abbreviations

ECR: European Congress of Radiology
ESR: European Society of Radiology
ESR-PAG: European Society of Radiology-Patients Advisory Group
UHC: Universal Health Care
VBHC: Value-Based Health Care
VBR: Value-Based Radiology
