# Supplementary material for: Patient survey of value in relation to radiology: results from a survey of the European Society of Radiology (ESR) value-based radiology subcommittee
Source: Insights Imaging. 2021 Jan 7;12:6. doi: 10.1186/s13244-020-00943-x (PMC7790952; doi:10.1186/s13244-020-00943-x)
Supplement: Supplementary file 1 — Additional file 1. English version of the survey and final set of data for analysis. [file 13244_2020_943_MOESM1_ESM.pdf]

Electronic Supplementary Material 1

Raw data from the survey. All tables correspond to the Figure with the same number.

Table 1: Responses by country

Country	Responses	
Australia	3	0.75%
Austria	84	21.00%
Belgium	8	2.00%
Bulgaria	15	3.75%
Canada	2	0.50%
Croatia	1	0.25%
Czech Republic	1	0.25%
France	158	39.50%
Germany	1	0.25%
Iraq	1	0.25%
Ireland	5	1.25%
Italy	3	0.75%
Liechtenstein	1	0.25%
Netherlands	18	4.50%
Nigeria	1	0.25%
Portugal	2	0.50%
Russian Federation	18	4.50%
Spain	9	2.25%
Switzerland	20	5.00%
Turkey	1	0.25%
United Kingdom	38	9.50%
United States of America	7	1.75%
**Answered**	**397**	
**Skipped**	**3**	
**TOTAL**	**400**	

Table 2:

Age	Printed Responses	Percentage	Online Responses	Percentage	Total (all responses)	Percentage
Under 20	3	1.20%	3	2.05%	6	1.50%
21-30	10	4.02%	9	6.16%	19	4.75%
31-40	15	6.02%	32	21.92%	47	11.75%
41-50	33	13.25%	43	29.45%	76	19.00%
51-60	64	25.70%	33	22.60%	97	24.25%
61-70	63	25.30%	18	12.33%	81	20.25%
71-80	50	20.08%	6	4.11%	56	14.00%
81-90	11	4.42%	2	1.37%	13	3.25%
91+	0	0.00%	0	0.00%	0	0.00%
**Total**	**249**		**146**		**395**	
				**Skipped**	**5**	
				**TOTAL**	**400**	

Table 3: Gender of respondents

Gender	Responses	
Female	255	63.75%
Male	137	34.25%
Other	1	0.25%
**Answered**	**393**	**98.25%**
**Skipped**	**7**	**1.75%**
**TOTAL**	**400**	

Table 4: How many times have you undergone radiological scans in the last two years?

Options	Responses	
0	4	1.00%
1-5	281	70.25%
6-10	81	20.25%
11-15	21	5.25%
16 or more	7	1.75%
**Answered**	**394**	**98.50%**
**Skipped**	**6**	**1.50%**
**TOTAL**	**400**	

Table 5: How satisfied were you with your overall experience with radiological services in the last two years?

Rating*	Responses	
1	9	2.25%
2	17	4.25%
3	40	10.00%
4	135	33.75%
5	188	47.00%
**TOTAL**	**389**	**97.25%**
**Skipped**	**11**	**2.75%**
**Avg rating**	**4.22**	

*(1= very unsatisfied, 5 = very satisfied)

Table 6: How satisfied were you with the information provided about the procedure (risks, benefits, description of how it is performed)?

Rating*	Responses	
1	24	6.00%
2	41	10.25%
3	44	11.00%
4	105	26.25%
5	86	21.50%
**TOTAL**	**300**	**75.00%**
**Skipped**	**100**	**25.00%**
**Avg rating**	**3.63**	

*(1= very unsatisfied, 5 = very satisfied)

Table 7: Were you supplied with a copy of the radiology report from your examination?

Options	Responses	
Yes	224	56.00%
No	154	38.50%
**Answered**	**378**	**94.50%**
**Skipped**	**22**	**5.50%**
**TOTAL**	**400**	

Table 8: How satisfied were you with the way your results were communicated to you?

Rating*	Responses	
1	23	5.75%
2	32	8.00%
3	63	15.75%
4	122	30.50%
5	116	29.00%
**TOTAL**	**356**	**89.00%**
**Skipped**	**44**	**11.00%**
**Avg rating**	**3.78**	

*(1= very unsatisfied, 5 = very satisfied)

Tables 9A-F: Please rate the following aspects of the radiology service (1 = very unsatisfied, 5 = very satisfied):

Table 9A: Courtesy of the staff

Rating	Frequency	Percent
**1**	8	2.00%
**2**	10	2.50%
**3**	43	10.75%
**4**	127	31.75%
**5**	194	48.50%
**Answered**	**382**	**95.50%**
**Skipped**	**18**	**4.50%**
**TOTAL**	**400**	
**Avg rating**	**4.28**	

Table 9B: Information provided about benefits & risks of the procedure

Rating	Frequency	Percent
**1**	37	9.25%
**2**	63	15.75%
**3**	72	18.00%
**4**	107	26.75%
**5**	88	22.00%
**Answered**	**367**	**91.75%**
**Skipped**	**33**	**8.25%**
**TOTAL**	**400**	
**Avg rating**	**3.40**	

Table 9C: Waiting times (i.e. from referral to appointment)

Rating	Frequency	Percent
**1**	15	3.75%
**2**	27	6.75%
**3**	77	19.25%
**4**	135	33.75%
**5**	118	29.50%
**Answered**	**372**	**93.00%**
**Skipped**	**28**	**7.00%**
**TOTAL**	400	
**Avg rating**	**3.84**	

Table 9D: Time-to-diagnosis (from initial referral)

Rating	Frequency	Percent
**1**	12	3.00%
**2**	26	6.50%
**3**	71	17.75%
**4**	136	34.00%
**5**	121	30.25%
**Answered**	**366**	**91.50%**
**Skipped**	**34**	**8.50%**
**TOTAL**	400	
**Avg rating**	**3.90**	

Table 9E: Information provided by radiology staff following diagnosis

Rating	Frequency	Percent
**1**	29	7.25%
**2**	34	8.50%
**3**	75	18.75%
**4**	104	26.00%
**5**	108	27.00%
**Answered**	**350**	**87.50%**
**Skipped**	**50**	**12.50%**
**TOTAL**	**400**	
**Avg rating**	**3.65**	

Table 9F: Availability of the radiologist for personal consultation

Rating	Frequency	Percent
**1**	51	12.75%
**2**	63	15.75%
**3**	64	16.00%
**4**	84	21.00%
**5**	83	20.75%
**Answered**	**345**	**86.25%**
**Skipped**	**55**	**13.75%**
**TOTAL**	400	
**Avg rating**	**3.25**	

Table 10: Were you previously familiar with the concept of value-based healthcare (VBH) / value-based radiology (VBR)?

Options	Responses	
Yes	71	17.75%
No	293	73.25%
**Answered**	**364**	**91.00%**
**Skipped**	**36**	**9.00%**
**Total**	**400**	

Table 11: Which do you consider the most important aspect of value in radiology?

Options	Frequency	Percentage
Cost (out of pocket or cost to the health system/insurer)	10	2.50%
Efficiency / safety	180	45.00%
Service (the way you are treated, waiting times, time-to-diagnosis)	122	30.50%
**Answered**	**312**	**78.00%**
**Skipped**	**68**	**17.00%**
**Gave multiple answers**		
Cost & Efficiency/Safety	2	0.50%
Cost & Service	4	1.00%
Efficiency/Safety & Service	14	3.50%
**TOTAL**	**400**	

Table 12: Which quality aspect do you consider most important in radiology?

	Ranking position*								
	1		2		3		4		
	**Frequency**	**Percentage**	**Frequency**	**Percentage**	**Frequency**	**Percentage**	**Frequency**	**Percentage**	**Average ranking***
Efficiency (the necessary results are obtained using fewest possible procedures / time)	21	*14.29%*	21	*14.29%*	21	*14.29%*	84	*57.14%*	**3.14**
Effectiveness (the correct examinations are performed to produce the necessary results for diagnosis)	43	*29.25%*	41	*27.89%*	51	*34.69%*	12	*8.16%*	**2.22**
Safety of the exams	28	*19.05%*	38	*25.85%*	44	*29.93%*	37	*25.17%*	**2.53**
Accuracy / correctness of the diagnosis	55	*37.41%*	47	*31.97%*	31	*21.09%*	14	*9.52%*	**2.03**
**TOTAL**	147		147		147		147		

*(1 = most important, 4 = least important)

rated as most important
rated as least important

Table 13: Which service aspect do you consider most important in radiology?

	Ranking position*												
	1		2		3		4		5		6		
	Frequency	Percentage	Frequency	Percentage	Frequency	Percentage	Frequency	Percentage	Frequency	Percentage	Frequency	Percentage	Average ranking*
Courtesy of the staff	17	*15.18%*	10	*8.93%*	19	*16.96%*	26	*23.21%*	16	*14.29%*	24	*21.43%*	3.76
Information provided about benefits & risks of the procedure	15	*13.39%*	14	*12.50%*	16	*14.29%*	23	*20.54%*	25	*22.32%*	19	*16.96%*	3.66
Waiting times (i.e. from referral to appointment)	24	*21.43%*	22	*19.64%*	17	*15.18%*	13	*11.61%*	21	*10.71%*	15	*13.39%*	3.27
Time-to-diagnosis	**27**	*24.11%*	24	*21.43%*	19	*16.96%*	16	*14.29%*	12		14	*12.50%*	**3.04**
Information provided following diagnosis by radiology staff	20	*17.86%*	15	*13.39%*	26	*23.21%*	22	*19.64%*	22	*19.64%*	7	*6.25%*	3.29
Availability of the radiologist for personal consultation	9	*8.04%*	27	*24.11%*	15	*13.39%*	12	*10.71%*	16	*14.29%*	**33**	*29.46%*	**3.88**
**TOTAL**	112		112		112		112		112		112		

*(1= most important, 6 = least important)

rated as most important
rated as least important

Table 14: What factors are most important to you in receiving radiology services? (Select the three most important to you).

Options	Responses	
	**Frequency***	**Percentage****
Appropriate scan is performed	107	39.78%
Being able to arrange an appointment online	6	2.23%
Being able to get an appointment quickly	55	20.45%
Diagnosis is quick	98	36.43%
Doctor consults patient on procedure options	40	14.87%
Doctor explains the procedure	39	14.50%
Doctor knows me / cares about me	25	9.29%
Doctors / nurses / staff are friendly	25	9.29%
My confidence in the doctor / their qualifications	94	34.94%
My confidence in the equipment used	37	13.75%
My health improves	38	14.13%
No errors are made in diagnosis	118	43.87%
Out-of-pocket expenses are affordable	5	1.86%
Radiologist is available to explain diagnosis	74	27.51%
Radiology facility is easily accessible	10	3.72%
Surroundings in hospital / scanner etc. are clean / pleasant	13	4.83%
Waiting times at the hospital / surgery etc. are short	22	8.18%
Other (please specify)	1	0.37%

**How many respondents selected that option in their top 3*

***% Equals percentage of 269 valid responses which included that option as one of their top three.*

ESR Value-Based Radiology Patient Survey

https://www.surveymonkey.com/r/2M6D3NV

The European Society of Radiology (ESR) is conducting a survey with the aim of better understanding what value in radiology means to patients. Completing this survey will help us work towards a future in which the field of radiology provides the greatest possible value to patients. The survey will take approximately 10 minutes to complete. We thank you for providing us with your input.

Part 1:

Country:

City:

Age:

Sex:

Part 2:

1. How many times have you undergone radiological scans in the last two years? (please tick)

0	1-5	6-10	11-15	16+
☐	☐	☐	☐	☐

2. What kind of imaging procedure(s) have you undergone in the last two years? (please tick)

- X-Ray ☐
- Computed Tomography (CT) ☐
- Magnetic Resonance Imaging (MRI) ☐
- Ultrasound (US) ☐
- Other ☐

3. How satisfied were you with your overall experience with radiological services in the last two years? (1= very unsatisfied, 5 = very satisfied) (please tick)

1	2	3	4	5
☐	☐	☐	☐	☐

4. Did you receive written information about the imaging procedure? (please tick)

- Yes ☐
- No ☐

5. How satisfied were you with the information provided about the procedure (risks, benefits, description of how it is performed)? (1= very unsatisfied, 5 = very satisfied) (please tick)

1	2	3	4	5
☐	☐	☐	☐	☐

6. Were you supplied with a copy of the radiology report from your examination? (please tick)

- Yes ☐
- No ☐

7. How satisfied were you with the way your results were communicated to you?
(1= very unsatisfied, 5 = very satisfied) (please tick)

1	2	3	4	5
☐	☐	☐	☐	☐

8. Are you aware that after undergoing an x-ray or CT procedure you are entitled to receive information about your radiation dose exposure? (please tick)

- Yes ☐
- No ☐

9. If you underwent x-ray or CT procedure, did you receive information about your radiation dose exposure after the procedure? (please tick)

- Yes ☐
- No ☐

10. Please rate the following aspects of the radiology service: (1= very unsatisfied, 5 = very satisfied) (please tick)

- Courtesy of the staff

1	2	3	4	5
☐	☐	☐	☐	☐

- Information provided about benefits & risks of the procedure

1	2	3	4	5
☐	☐	☐	☐	☐

- Waiting times (i.e. from referral to appointment)

1	2	3	4	5
☐	☐	☐	☐	☐

- Time to diagnosis (i.e. from initial referral)

1	2	3	4	5
☐	☐	☐	☐	☐

- Information provided following diagnosis by radiology staff

1	2	3	4	5
☐	☐	☐	☐	☐

- Availability of the radiologist for personal consultation

1	2	3	4	5
☐	☐	☐	☐	☐

11. What could have improved your experience?

Part 3:

12. Value-based care is a philosophy of healthcare achieved when professionals intentionally consider the quality of care provided, and the overall outcomes of that care, in relation to cost-efficiency.

Were you previously familiar with the meaning of value-based healthcare (VBH) / value-based radiology (VBR)? (please tick)

- Yes ☐
- No ☐

13. Which do you consider the most important aspect of value in radiology? (please tick)

- Cost (out of pocket or cost to the health system/insurer) (go to **part 4**) ☐
- Efficiency / Safety (go to **part 5**) ☐
- Service (the way you are treated, waiting times, time-to-diagnosis) (go to **part 6**) ☐

Part 4: if cost:

14. Do you have public/statutory/mandatory or private health insurance? (please tick)

- Public ☐
- Private ☐
- Both ☐
- Neither ☐

Go to part 7

Part 5: if efficiency / safety:

15. Which aspect of efficiency/safety do you consider most important in radiology? (please rank them according to importance) (1 = most important, 4 = least important)

- Efficiency (the necessary results are obtained using fewest procedures/time) ☐
- Effectiveness (the correct examinations are performed to produce the necessary results for diagnosis) ☐
- Safety of the exams ☐
- Accuracy/correctness of the diagnosis ☐

Go to part 7

Part 6: if service:

16. Which service aspect do you consider most important in radiology? (please rank them according to importance) (1 = most important, 6 = least important)

- Courtesy of the staff ☐
- Information provided about benefits & risks of the procedure ☐
- Waiting times (i.e. from referral to appointment) ☐
- Time to diagnosis ☐
- Information provided following diagnosis by radiology staff ☐
- Availability of the radiologist for personal consultation ☐

Go to part 7

Part 7:

17. Which factor do you think most affects the value of radiology within the healthcare chain? (please tick)

- Referral ☐
- Treatment decisions based on diagnosis ☐
- Follow up / monitoring ☐

18. Is cost or quality of service more important in defining 'value'? (please tick)

- Cost ☐
- Quality ☐
- Both ☐

19. Are you able to choose where you receive radiology services? (please tick)

- Yes (go to **question 20**) ☐
- No (go to **question 21**) ☐

20. Which of the following is most important to you when you choose where you receive radiology services? (please tick)

- Cost (the amount I pay for the services I receive) ☐
- Quality (the efficiency, effectiveness, safety, and results or outcomes of the care I receive) ☐
- Service (the way I am treated as a patient) ☐
- Location (close to home/work, accessible) ☐

21. What factors are most important to you in receiving radiology services? (please select the three most important to you). (1 = most important, 3 = least important)

- Appropriate scan is performed ☐
- Being able to arrange an appointment online ☐
- Being able to get an appointment quickly ☐
- Diagnosis is quick ☐
- Doctor consults patient on procedure options ☐
- Doctor explains the procedure ☐
- Doctor knows me / cares about me ☐
- Doctors / nurses / staff are friendly ☐
- My confidence in the doctor / their qualifications ☐
- My confidence in the equipment used ☐
- My health improves ☐
- No errors are made in diagnosis ☐
- Out-of-pocket expenses are affordable ☐
- Radiologist is available to explain diagnosis ☐
- Radiology facility is easily accessible ☐
- Surroundings in hospital / scanner etc. are clean / pleasant ☐
- Waiting times at the radiology facility are short ☐
- Other ☐

End of survey

Thank you for providing us with your feedback!

ESR EUROPEAN SOCIETY OF RADIOLOGY
